# Genome sequence and phenotypic analysis of a first German *Francisella sp*. isolate (W12-1067) not belonging to the species *Francisella tularensis*

**DOI:** 10.1186/1471-2180-14-169

**Published:** 2014-06-25

**Authors:** Kerstin Rydzewski, Tino Schulz, Elzbieta Brzuszkiewicz, Gudrun Holland, Christian Lück, Jens Fleischer, Roland Grunow, Klaus Heuner

**Affiliations:** 1 Cellular Interactions of Bacterial Pathogens, Centre for Biological Threats and Special Pathogens, Division 2 (ZBS 2), Robert Koch Institute, Nordufer 20, Berlin 13353, Germany; 2 Department of Genomics and Applied Microbiology & Göttinger Genomics Laboratory, Institute of Microbiology and Genetics, Georg-August-University of Göttingen, Grisebachstr. 8, Göttingen 37077, Germany; 3 Centre for Biological Threats and Special Pathogens, Division 4 (ZBS 4), Advanced Light and Electron Microscopy, Robert Koch Institute, Nordufer 20, Berlin 13353, Germany; 4 Institute of Medical Microbiology and Hygiene, Consultant Laboratory for Legionella, TU Dresden, Fiedlerstr. 42, Dresden 01307, Germany; 5 Landesgesundheitsamt Baden-Württemberg, Nordbahnhof 135, Stuttgart 70191, Germany; 6 Centre for Biological Threats and Special Pathogens, Division 2 (ZBS 2), Highly Pathogenic Microorganisms, Robert Koch Institute, Nordufer 20, Berlin 13353, Germany

**Keywords:** Francisella isolate, W12-1067, Cooling tower, Genome sequence, Pathogenicity island FPI, Environmental, Legionella, Germany

## Abstract

**Background:**

*Francisella* isolates from patients suffering from tularemia in Germany are generally strains of the species *F. tularensis* subsp. *holarctica*. To our knowledge, no other *Francisella* species are known for Germany. Recently, a new *Francisella* species could be isolated from a water reservoir of a cooling tower in Germany.

**Results:**

We identified a *Francisella* sp. (isolate W12-1067) whose 16S rDNA is 99% identical to the respective nucleotide sequence of the recently published strain *F. guangzhouensis*. The overall sequence identity of the *fopA*, *gyrA*, *rpoA*, *groEL*, *sdhA* and *dnaK* genes is only 89%, indicating that strain W12-1067 is not identical to *F. guangzhouensis*. W12-1067 was isolated from a water reservoir of a cooling tower of a hospital in Germany. The growth optimum of the isolate is approximately 30°C, it can grow in the presence of 4–5% NaCl (halotolerant) and is able to grow without additional cysteine within the medium. The strain was able to replicate within a mouse-derived macrophage-like cell line. The whole genome of the strain was sequenced (~1.7 mbp, 32.2% G + C content) and the draft genome was annotated. Various virulence genes common to the genus *Francisella* are present, but the *Francisella* pathogenicity island (FPI) is missing. However, another putative type-VI secretion system is present within the genome of strain W12-1067.

**Conclusions:**

Isolate W12-1067 is closely related to the recently described *F. guangzhouensis* species and it replicates within eukaryotic host cells. Since W12-1067 exhibits a putative new type-VI secretion system and *F. tularensis* subsp. *holarctica* was found not to be the sole species in Germany, the new isolate is an interesting species to be analyzed in more detail. Further research is needed to investigate the epidemiology, ecology and pathogenicity of *Francisella* species present in Germany.

## Background

*Francisella tularensis* is an facultative intracellular pathogen that causes tularemia in humans and a wide range of animals [1]. Strains of *F. tularensis* subsp. (*Ft.*) *tularensis* can be lethal to humans and are mostly associated with cases of tularemia in the U.S.A. Doses as low as 10–20 bacteria can be infective [1]. Transmission mostly occurs via aerosol, alimentary ingestion or skin inoculation. In addition, *F. tularensis* is suspected as a potential bacterial biological weapon [2]. The species *Ft. novicida* is almost avirulent for humans in contrast to mice, and is thought to be an opportunistic pathogen [3,4]. *Ft. novicida* is assumed to constitute an environmental lineage along with *F. philomiragia*. In rare cases the latter has also been associated with human disease in immunocompromised individuals [4,5].

Human infections caused by *F. tularensis* are rare in Germany, but seroprevalence studies in wild animals revealed a high seroprevalence of *F. tularensis* in wildlife in eastern Germany [6-8]. In Germany, *Ft. holarctica* is generally identified in affected animals or humans as well as in known vectors (like ticks and other arthropods) [1,9-11]. Other as yet known species of the genus *Francisella* are *F. hispaniensis*[12,13], *F. halioticida*[14], *F. piscida*[15], *F. noatunensis*[16], *F. asiatica*[16], *F. noatunensis* subsp. *orientalis*[17] and *F. philomiragia* subsp. *noatunensis*[18,19]. Very recently, a new *Francisella* species (*F. guangzhouensis*) was isolated from a cooling tower in China, which had not been reported before [20].

However, to our knowledge, no species other than *Ft. holarctica* has been identified in Germany until now. Therefore, our new isolate W12-1067 is the first aquatic isolate identified in Germany which does not belong to the species *F. tularensis* and is closest related to *F. guangzhouensis*.

## Methods

### Strains, media and growth conditions

Strains used in this study were *Ft* subsp. *holarctica* LVS (ATCC 29684), *Ft. novicida* U112 (ATCC 15482), *F. philomiragia* (ATCC 25015), *Legionella pneumophila* Paris (CIP 107629) and the new environmental *Francisella* isolate W12-1067. *Francisella* strains were cultivated in medium T [21] (1% brain heart infusion broth [Difco Laboratories, Inc., Sparks, MD, USA], 1% bacto tryptone [Difco], 1% technical casamino acids [Difco], 0.005 g of MgSO_4_, 0.01% FeSO_4_, 0.12% sodium citrate, 0.02% KCl, 0.04% K_2_HPO_4_, 0.06% L-cysteine and 1.5% glucose) or on enriched cystine-heart agar (CHA [Difco], 1% brain heart infusion broth, 1% proteose-peptone, 1% D-glucose, 0.5% NaCl, 0.1% L-cystine, 1.5% agar and 1% hemoglobin). W12-1067 and *L. pneumophila* Paris were cultivated in ACES-buffered yeast extract (AYE) broth [1% N-(2-acetamido)-2-aminoethanesulfonic acid (ACES), 1% yeast extract, 0.04% L-cysteine and 0.025% ferric pyrophosphate, adjusted to pH 6.8 with 3 M potassium hydroxide (KOH) and sterile filtrated], on ACES-buffered charcoal–yeast extract (BCYE) agar [22] or on GVPC agar plates (Heipha Dr. Müller GmbH, Eppelheim, Germany, BCYE agar supplemented with 80,000 IE polymyxin B, 1 mg/l of vancomycin and 80 mg/l of cycloheximide). Isolate W12-1067 was initially cultivated on GVPC agar plates. The U937 human macrophage-like cell line ATCC CRL-1593.2 and the mouse macrophage cell line J774A.1 were cultivated in RPMI 1640 + 10% FCS medium (PAA/GE Healthcare Europe GmbH, Freiburg, Germany) at 37°C and 5% CO_2_.

### Phenotypic assays

Growth without additional cysteine was done on BCYE agar plates without additional cysteine. Physiological characteristics of analyzed strains were determined by using API ZYM (bioMérieux Deutschland GmbH, Nürtingen, Germany).

Chitinase activity tests were done on 1.5% agarose plates containing 0.1% deacetylated glycol chitin. Chitinase activity experiments were done as described earlier [23], with the modification that the deacetylated glycol chitin was suspended in 0.01 M sodium phosphate (pH 5.5) by heating. In short, bacteria were grown in medium T overnight. The supernatant was precipitated by isopropanol and the protein pellet was resuspended in PBS (concentrated 40-fold). 50 μl were inoculated into agar plates (as described earlier [24]) and incubated for two days at 37°C. Degrading activity was visualized by incubation with 0.01% Calcofluor Brightener 28 (Sigma-Aldrich Chemie GmbH, Munich, Germany) for 10 min, washing two times with water and then incubation at room temperature (RT) overnight.

The NaCl sensitivity assays were done in 96-well plates in a total volume of 200 μl of medium T and ~2 × 10^7^ bacterial cells. Plates were incubated 2–3 days at 37°C and 5% CO_2_, and then the optical density (OD) at 600 nm was measured using an Infinite 200 reader (Tecan Deutschland GmbH, Crailsheim, Germany).

### Intracellular multiplication of *Francisella* in host cells

To determine which amoeba strain may be suitable to be used for replication assays, isolate W12-1067 (~10^10^ cells) was suspended in 1 ml of dH_2_O and 100 μl were plated onto NN-agar plates (14 g/l of agar in dH_2_O). The amoeba strain (15 μl, *A. castellanii* ATCC 30010, *A. castellanii* ATCC 30234, *A. castellanii* 50739, *A. lenticulata* 45 ATCC 50703, *A. lenticulata* 118 ATCC 50706, *Hartmannella vermiformis* OS101, *Hartmannella vermiformis* ATCC 50256 and *Naegleria gruberi* ATCC 30244, respectively) was dropped onto the centre of the plates and incubated at RT or at 30°C for 7 days. The plates were inspected daily for movement and replication of the amoeba. All amoeba tested were motile and not killed by isolate W12-1067. Therefore, no further experiments were performed using amoebae as host cells.

For differentiation into macrophage-like cells, U937 cells were adjusted to 3 × 10^5^ cells/ml and transferred into 100 ml of fresh RPMI medium containing 10% fetal calf serum (10% FCS), and PMA (phorbol-12-myristate-13-acetate, 1 mg/ml in dH_2_O [P-8139; Sigma-Aldrich Chemie]) was added at a concentration of 1:20,000. After incubation for 36 h at 37°C and 5% CO_2_, the supernatant was discarded and adherent cells were washed once with 10 ml of 0.2% EDTA in PBS. Cells were mechanically detached from the flask bottom with RPMI + 10% FCS, transferred into 50 ml tubes and centrifuged at 800 g for 10 min. All cells were counted after trypan blue staining in a Neubauer counting chamber and adjusted to 5 × 10^5^ cells/ml with RPMI + 10% FCS. To each well of a 24-well plate 1 ml of the cell suspension was added and incubated for adhesion during 2 h at 37°C and 5% CO_2_. Macrophage-like mouse cell line J774A.1 was also grown in fresh RPMI medium containing 10% fetal calf serum and treated as described above, but without the differentiation step (no PMA treatment).

For both cell lines, stationary phase bacteria grown for 3 days on CHA or BCYE agar were diluted in plain RPMI medium and the infection was done with a multiplicity of infection (MOI) of 1, 10 or 100 (time point 0 h) for 2 h at 37°C and 5% CO_2_. Cells were washed three times with RPMI and incubated with 50 μg/ml of Gentamycin for 1 h to kill extracellular bacteria. Cells were washed again three times with RPMI and covered with 1 ml of RPMI + 10% FCS. For colony-forming unit (CFU) determination at various time points of infection, coincubations of cells and bacteria were lysed by addition of 10 μl of 10% Saponin (S4521, Sigma-Aldrich Chemie) for 5 min, and serial dilutions were plated on BCYE agar. In a control experiment we showed that Saponin treatment did not affect the number of remaining CFU of strain W12-1067 (data not shown).

### Electron microscopy (EM)

J774A.1 cells were infected with *Francisella* strain W12-1067 (MOI of 10) at 37°C as described above. Cells were fixed 96 h post infection with 2.5% glutaraldehyde in 0.05 M HEPES buffer. Bacteria cultivated in medium T were fixed with 4% paraformaldehyde 5% glutaraldehyde in 0.05 M HEPES buffer for 2 h at RT. All samples were post-fixed with osmium tetroxide (1% in distilled water) and uranyl acetate (2% in distilled water), dehydrated stepwise in a graded ethanol series and embedded in LR White resin (Science Services GmbH, Munich, Germany) which was polymerized at 60°C overnight. Thin sections were prepared with an ultramicrotome (UC-T; Leica, Vienna, Austria) and counterstained with uranyl acetate and lead citrate.

Samples were examined using a transmission electron microscope (EM 902; Carl Zeiss Microscopy GmbH, Jena, Germany) at 80 kV, and the images were recorded using a slow-scan charge-coupled-device camera (Pro Scan elektronische Systeme GmbH, Lagerlechfeld, Germany).

### Genome sequencing, ORF finding and annotation

Genome sequencing of chromosomal DNA of isolate W12-1067 was performed by Eurofins MWG Operon (Eurofins Medigenomix GmbH, Ebersberg, Germany): (i) Short insert shotgun library (FLX + library): 1 μg of DNA was fragmented using a Covaris E210 instrument (Covaris Inc., Woburn, MA) according to manufacturer’s instructions. End-repair, dA-tailing and ligation of barcoded adapter were performed following New England Biolabs’ instructions (New England Biolabs GmbH, Frankfurt/Main, Germany). Emulsion-based clonal amplification (emPCR amplification) was performed following Roche’s instructions (Roche Diagnostics GmbH, Mannheim, Germany). FLX + Sequencing: Sequencing was performed on an FLX + platform according the manufacturer’s instructions using 1/8 plate. The sequencing process was controlled by the Roche 454 software gsRunProcessor v2.8 (shotgun signal processing pipeline). (ii) 8 kb mate-pair-like library (Long-Jumping-Distance library): Creation of the 8 kb mate-pair-like library was done at Eurofins MWG Operon (Ebersberg, Germany) using their proprietary protocol. (iii) Illumina Sequencing: For sequencing, the library was loaded on an Illumina MiSeq machine. Cluster generation and paired-end sequencing was performed using the manufacturer’s instructions. MiSeq Control Software 2.2.0 was used for sequencing. For processing of raw data RTA version 1.17.28 and CASAVA 1.8.2 were used to generate FASTQ-files. (iv) Data analysis: A two-step hybrid de novo assembly was conducted using the sequencing data of the two libraries. First, the FLX + data (long reds, single-end) has been assembled separately using the Roche 454 software Newbler (v2.6). The resulting contigs as well as the Illumina long-jumping-distance pairs (Illumina mate-pair-like) were then assembled together with a hardware-accelerated assembly pipeline based on the Convey hardware and software tools (http://www.conveycomputer.com) that mimic a standard de novo assembly using the Velvet assembler (Eurofins proprietary assembly pipeline) [25,26]. The draft genome (all contigs) was annotated by using the RAST server, freely available at http://www.patricbrc.org[27].

### Phylogenetic analysis

16S rDNA gene and the multi-gene locus (in frame gene sequence) of genes *fopA*, *gyrA*, *rpoA*, *groEL*, *sdhA* and *dnaK* of strain W12-1067 and available homologous sequences from *Francisella* species and *L. pneumophila* Paris (obtained from GenBank) were used for nucleotide comparison. The multi-gene locus of *L. pneumophila* Paris exhibited no *fopA* gene because no homolog is present within the genome. Phylogenetic analysis (phylogenetic tree) was generated by using the ClustalW program (ClustalW 2.1; http://www.patricbrc.org).

### Accession number

This whole genome shotgun project has been deposited at DDB/EMBL/GenBank under accession AWHF00000000. The version described in this paper is version [AWHF01000000].

## Results and discussion

During 2012 health authorities of the city of Heilbronn (Germany) observed some coincident spots of Legionnaires’ disease (LD). Therefore, different putative sources were screened for the presence of *Legionella* species using GVPC (glycine-vancomycin-polymyxin-cycloheximide) agar plates. The investigation of a water reservoir of a cooling tower led to the isolation of strain W12-1067. The isolate W12-1067 was first thought to belong to the genus *Legionella* because of its habitus and growth on GVPC agar plates, but PCR analysis did not support this finding. Preliminary 16S rDNA PCR analysis performed by the German Consultant Laboratory for Legionella (Dresden, Germany) revealed that this strain may belong to the genus *Francisella*. The identified isolate was not involved in the LD outbreak. However, the strain was send for further analysis to the Centre for Biological Threats and Special Pathogens at the Robert Koch Institute (Berlin, Germany). Here, W12-1067 was identified to be the first German isolate of the genus *Francisella* which did not belong to the species *F. tularensis*.

### Analysis of strain W12-1067

**Figure 1:**
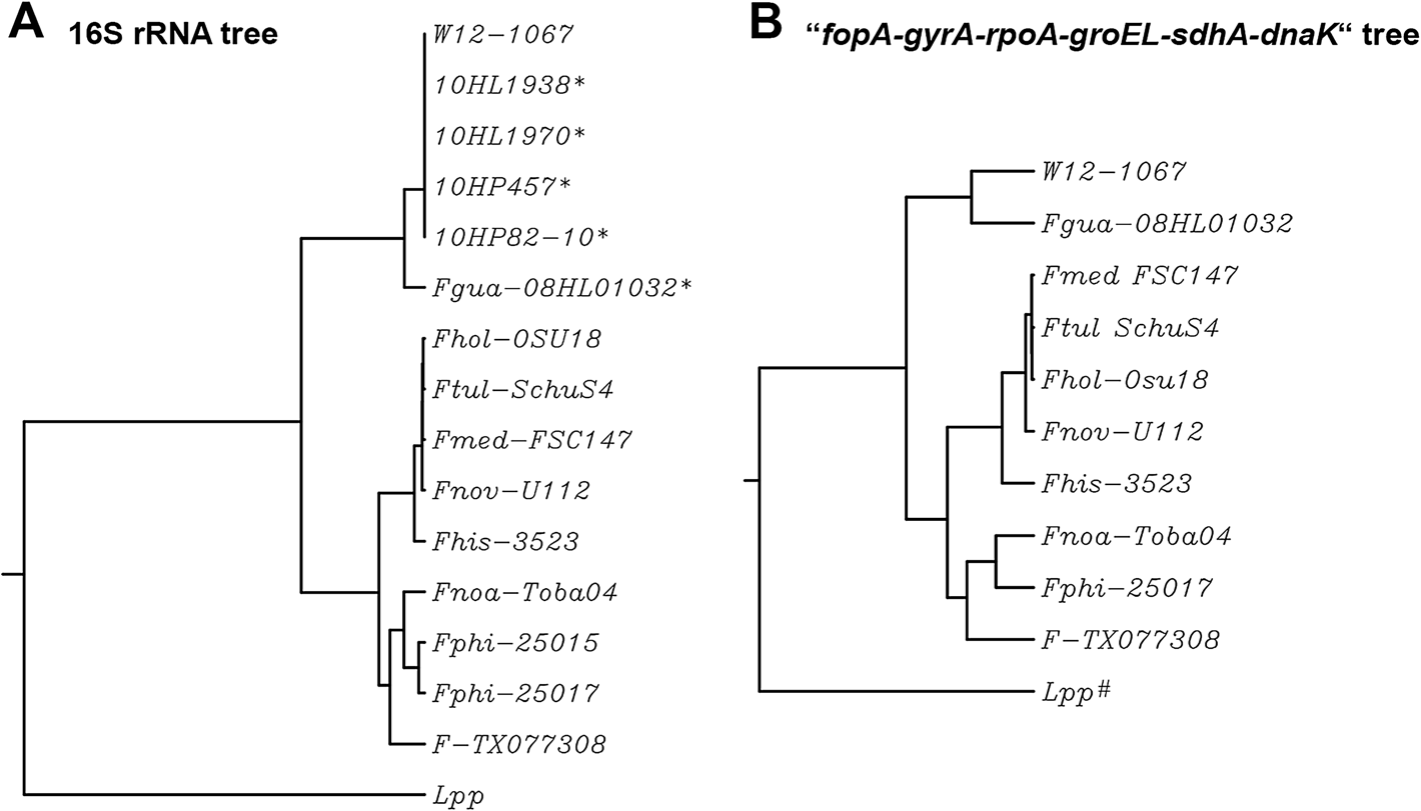
**Phylogenetic tree analysis. (A)** Phylogenetic tree analysis of different *Francisella* strains using 16S rDNA or **(B)** 6-loci concatenated DNA sequences. Name and function of genes used for the 6-loci concatenated sequences are given in Table 2. *, 16S rDNA sequences of different isolates of *F. guangzhouensis* published by [20]; #, Concatenated sequence of *L. pneumophila* Paris (Lpp) did not exhibit a *fopA* gene, because no homolog of this gene is present within the genome sequence. Fhol-OSU18, *Ft. holartica* strain OSU18; Ftul-SchuS4, *Ft. tularensis* strain SchuS4; Fmed-FSC147, *F. mediasiatica* strain FSC147, Fnov-U112, *Ft. novicida* strain U112; Fhis-3523, *F. hispaniensis* (*Ft. novicida*-like strain 3523); Fnoa-Toba04, *F. noatunensis* strain Toba04; Fphi-25015 and 25017, *F. philomiragia* strain ATCC 25015 and ATCC 25017; F-TX077308, *Francisella* isolate TX077308.

First we performed 16S rDNA PCR and sequenced the PCR product. The phylogenetic analysis of the 16S rDNA revealed that isolate W12-1067 is a close representative of the recently identified new environmental *Francisella* species *F. guangzhouensis*[20]. The phylogenetic tree of 16S rDNA of different *Francisella* strains is given in Figure 1A, corroborating the close relationship of isolate W12-1067 with *F. guangzhouensis* and other Chinese cooling tower isolates of this species (99% identity). The other *Francisella* strains analyzed revealed DNA identities of 16S rDNA sequences of 94–95% with 16S rDNA of isolate W12-1067 and 83% identity with the 16S rRNA gene of *L. pneumophila* Paris.

We then sequenced the whole genome of W12-1067, resulting in an annotated draft genome of this strain. We used the sequences of the genes *fopA*, *gyrA*, *rpoA*, *groEL*, s*dhA* and *dnaK* (see Table 1) to build a multi-gene locus (9918 bp for strain W12-1067) for each strain analyzed. With these 6-loci concatenated sequences we performed a further phylogenetic analysis (Figure 1B). The overall DNA identity was only 89%, indicating that W12-1067 is not identical to strain *F. guangzhouensis,* but that *F. guangzhouensis* is the closest relative identified to date. The overall DNA identity of the gene cluster of isolate W12-1067 to other *Francisella* strains analyzed was 80–81% and that to *L. pneumophila* Paris 67% (Figure 1B).

**Table 1:** Genes used for phylogenetic tree analysis

**Gene name**	**Peg Nr.**	**Feature**	**% DNA similarity to *F. gua*-08HL01032**^**T**^
16S rRNA	-	Small subunit ribosomal RNA	99%
23S rRNA	-	Large subunit ribosomal RNA	98%
*fopA*	25	*Francisella* outer membrane protein A	87%
*gyrB*	513	DNA gyrase subunit B	88%
*rpoA*	853	DNA-directed RNA polymerase A	90%
*groEL*	1260	Heat shock protein 60, chaperone	91%
*sdhA*	1002	Succinate dehydrogenase subunit A	88%
*dnaK*	687	Heat shock protein K, chaperone	92%

Type strain of *F. guangzhouensis* sp. nov (Qu et al. [20]).

Similar to strain W12-1067, *F. guangzhouensis* strains had been isolated from water of air conditioning systems of cooling towers in China, during a routine investigation to detect *Legionella*[20]. The growth optimum of this species ranged between 25 and 28°C and it showed growth on BCYE-alpha (minus cysteine) *Legionella*-agar plates. Furthermore, no virulence to mice was found for this strain [20,28]. No further information about virulence properties of this species was available yet.

**Figure 2:**
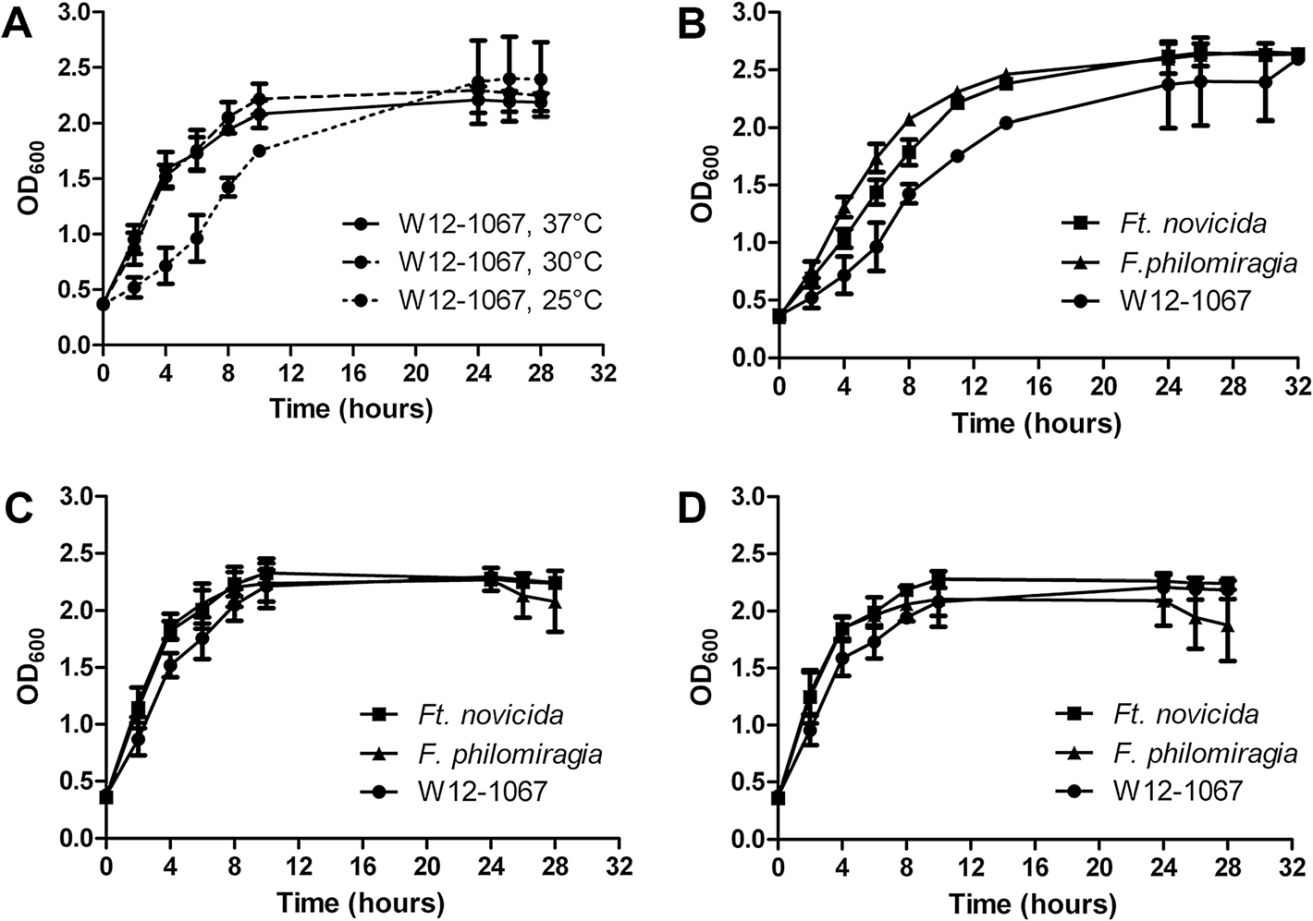
**Growth of different *Francisella *strains at 25, 30 and 37°C in medium T. (A)** Comparison of growth of *Francisella* isolate W12-1067 at different growth temperatures. **(B–D)** Comparison of growth of *Francisella* isolate W12-1067 with the growth of strains *Ft. novicida* U112 (*Ft. novicida*) and *F. philomiragia* 25015 (*F. philomiragia* ATCC 25015) at 25°C **(B)**, 30°C **(C)** and 37°C **(D)**. Results are mean standard deviations of three independent experiments of duplicate samples.

We investigated the growth of strain W12-1067 on different agar plates and within different liquid media. *Francisella* sp. strain W12-1067 grew well on BCYE, GVPC and CHA plates. The isolate grew faster in medium T than in AYE medium, and it did not grow in the cell culture medium RPMI (data not shown). Whereas L-cysteine within BCYE agar plates is necessary for the growth of *L. pneumophila* Paris, it stimulates the growth of *F. philomiragia*, and the growth of strain W12-1067 was nearly similar with or without additional cysteine (see Additional file 1: Figure S1A). Growth in medium T revealed that the growth optimum of strain W12-1067 is about 30°C (Figure 2A), but growth of strains *F. philomiragia* and *Ft. novicida* in general was faster and reached a higher cell density compared to that of the growth of strain W12-1067 (Figure 2B–D). Growth of strain W12-1067 in media with NaCl was reduced from a concentration of 4–5% NaCl, which was comparable to growth of strains *F. philomiragia* and *Ft. novicida* (up to 6–7% NaCl), but more resistant than the non-halotolerant strain *Ft. holarctica* LVS (see Additional file 1: Figure S1B). Using the API ZYM assay kit (bioMérieux), W12-1067 showed a profile similar to *F. guangzhouensis* (data not shown). In contrast to *F. guangzhouensis*, isolate W12-1067 was negative for alkaline phosphatase activity and showed only very low activity of the acid phosphatase, which is in good agreement with only one putative phosphatase encoding gene (peg_768) present within the genome sequence (see below).

**Figure 3:**
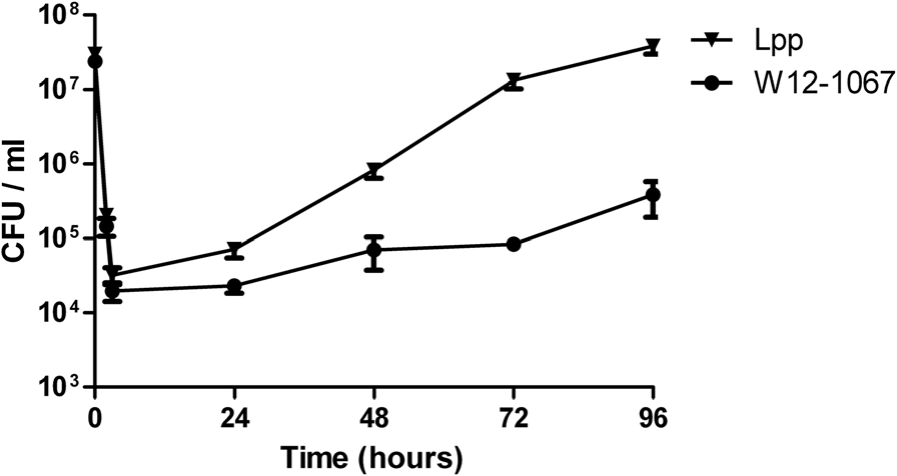
**Infection assays of *Francisella *strain W12-1067 and *L. pneumophila *Paris (Lpp) using the J774A.1 mouse cell line.** Growth curve over a period of four days. Cells were infected with bacteria at an MOI of 10. Cells were washed three times with RPMI and incubated with 50 μg/ml of Gentamycin for 1 h to kill extracellular bacteria. Cells were washed again three times with RPMI and covered with 1 ml of RPMI + 10% FCS. The number of CFU per well was determined by plating on CHA agar plates. Results are mean standard deviations of three independent experiments of duplicate samples.

**Figure 4:**
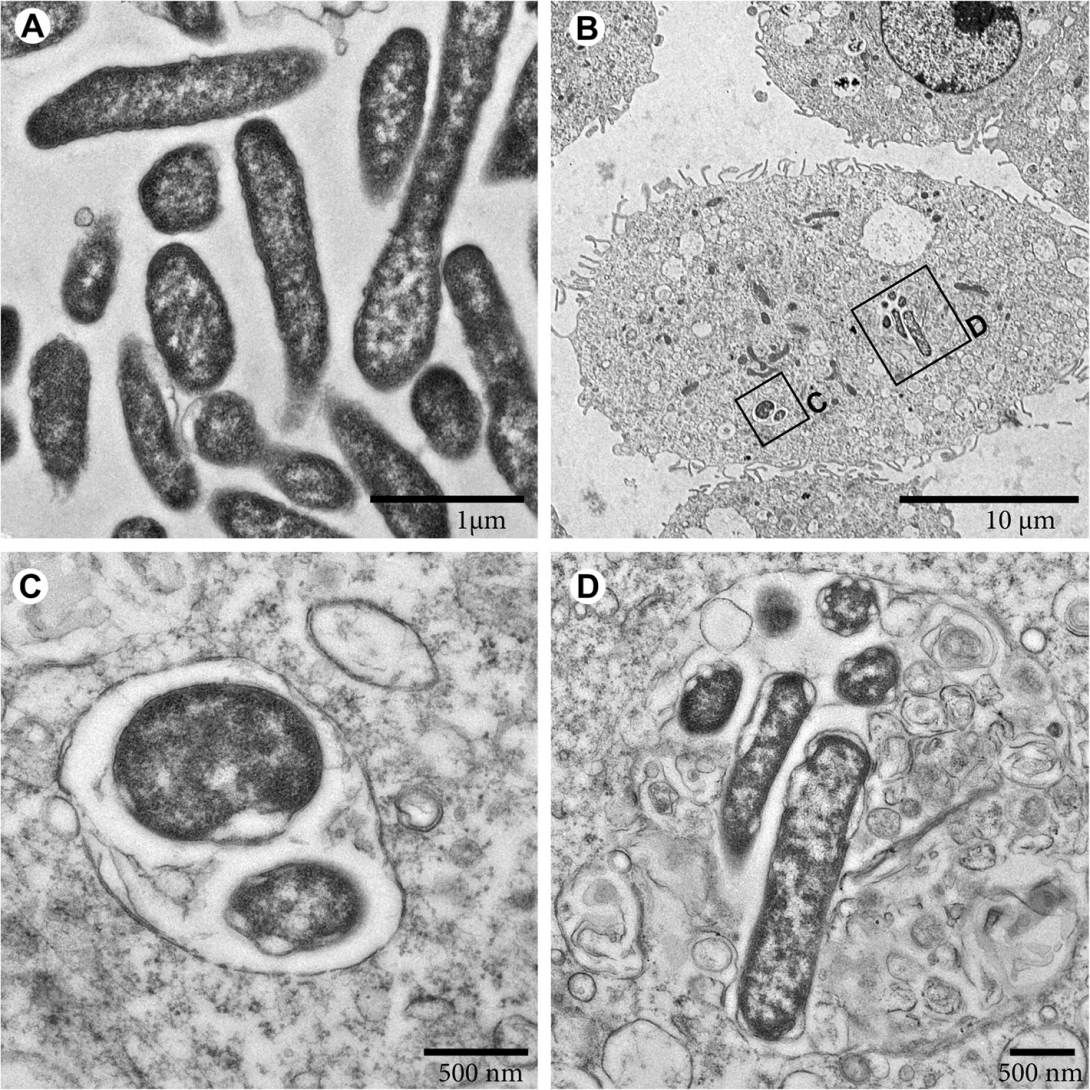
**Thin-section EM of *Francisella *strain W12-1067. (A)** Bacteria cultivated in medium T at 37°C possess a rod-shaped, slightly pleomorphic morphology. **(B-D)** Incubation of J774A.1 cells with bacteria (MOI 10). **(B)** Overview of host cells with two compartments containing bacteria (96 h post infection). Rectangles mark regions shown at higher magnification in C and D, respectively. **(C)** Two bacteria in a membrane-bound compartment. **(D)** Several bacteria in a compartment which shows a clearly discernible membrane at least in some regions.

These experiments were followed by co-culture studies using macrophage-like cell lines of human (U937) or mice (J774A.1) origin as host cells. We found that strain W12-1067 was able to persist within U937 cells (data not shown), and it replicated intracellularly in J774A.1 cells (Figure 3). Replication within J774A.1 was slower than that of *L. pneumophila* Paris. However, W12-1067 multiplied about 16-fold within 4 days of co-incubation, with a 24 h lag-phase at the beginning of the infection (Figure 3), indicating that the new isolate is able to infect and multiply within eukaryotic cells. Cells of strain W12-1067 grown in medium T at 37°C possessed a rod-shaped, slightly pleomorphic morphology (Figure 4A). EM analysis of the infection assay revealed that strain W12-1067 was localized intracellularly within a vacuole 96 h after infection (Figure 4B–D). The data indicate that W12-1067 is able to replicate intracellularly, but from EM analysis it was not clear yet whether the bacteria were able to escape from the vacuole. Experiments are under way to investigate this question further.

### Whole genome sequencing and sequence analysis of strain W12-1067

#### General features

The genomic DNA was sequenced using 454 shotgun reads and paired-end Illumina reads (for details, see Methods), followed by *de novo* assembly of the sequence, which was done by Eurofins (Ebersberg, Germany), resulting in 73 contigs and a chromosomal size of approximately 1,704,745 bp. The draft genome was annotated by using the RAST server (freely available at http://rast.nmpdr.otg). All genes encoded by the draft genome sequence of isolate W12-1067 (GenBank AWHF01000000) are given in the Additional file 2: Table S1. The draft genome exhibits 1,541 protein-coding genes (*peg*), three copies of the 16S-23S-5S rRNA gene locus (plus one additional 5S rRNA gene) and 37 tRNA genes (Table 2). The G + C content is 32.2% and therefore not significantly different from various other *Francisella* strains already sequenced [12,29-37]. However, the number of protein-coding genes and of tRNA genes seemed to be lower compared with other *Francisella* strains (Table 2), but this is not surprising since the W12-1067 genome is of draft quality and the others are finished. The genes encoding the tRNAs are present in four (tRNA-Leu), three (tRNA-Ala, -Ile, -Met, -Arg, -Gly, -Ser) and two (tRNA-Val, -Thr) copies. Like in other *Francisella* genomes, W12-1067 genome exhibits only one copy of the other tRNA genes (Asn, Asp, Cys, Gln, Glu, His, Lys, Phe, Pro, Trp and Tyr). The tRNA genes for Ala and Ile are only present within the ribosomal RNA locus, which is also true for the other *Francisella* strains and for most other bacteria [12,31,38,39]. Genome sequence comparison using the MAUVE program (http://www.DNASTAR.com) demonstrated that the genome of strain W12-1067 is poorly similar to the genomes of *Ft. holarctica*, *Ft. novicida*, *F. philomiragia* or *F. hispaniensis* AS02-814 (*Ft. novicida-like 3523*), indicating the evolutionary distance between these strains (data not shown). The MAUVE alignment of the draft genome sequence of strain W12-1067 with the genome sequence of *Ft. novicida* U112 is shown in the Additional file 3: Figure S2. The genome sequence of strain *F. guangzhouensis* 08HL01032^T^ was not yet available for a comparative analysis, but a G + C content of about 32.5% of its draft genome was reported [20].

**Table 2:** **Genome features of various *Francisella *** **genome sequences**

**Genome feature**	W12-1067*	***Fnov*-U112**	***Fphi*-25017**	***F-TX077308***
Chromosome size (bp)	1,704,745	1,910,031	2,045,775	2,035,931
Nr. of protein-coding genes	1,541	1,733	1,915	1,976
GC content (%)	32.2	32.48	32.57	32.9
5S rRNA genes	3 + 1	3 + 1	3 + 1	3 + 1
16S RNA genes	3	3	3	3
23S RNA genes	3	3	3	3
tRNA genes	37	39	39	39
GenBank accession number	AWHF01000000	CP000439	CP000937	CP002872

*Draft genome (73 contigs); Fnov-U112, *F. tularensis* subsp. *novicida* U112; Fphi-25017, *F. philomiragia* strain ATCC25017; F-TX077308, *Francisella spp*. strain TX077308.

#### Mobile elements

In *F. tularensis* strains six different insertion sequences (IS) elements (ISFtu1–ISFtu6) have been described. IS elements are important elements of *F. tularensis* genomes and are thought to be generally stable among different isolates [36,40]. Within the genome of *Francisella* isolate W12-1067, we identified several mobile elements and transposases (Table 3). For the element ISFw3 (1,177 bp in length) we identified 18 copies within the genome sequence. The element encodes a putative integrase of 359 amino acids with 70% amino acid identity to the protein encoded by the IS481 element of *Wolbachia* endosymbiont of *Drosophila simulans*. All other elements are only present as a single copy. There is only one element (ISFw7) with significant similarity to one of the ISFtu elements of *F. tularensis*. ISFw7 encodes a putative transposase exhibiting 78% amino acid identity to ISFtu1 of *Ft. holarctica* strains. Of the 14 mobile elements identified, six (ISFw4, 5, 7, 9, 10 and peg_1255) exhibit homologs within *Francisella* strains (Table 3).

**Table 3:** **Mobile elements and transposases of *Francisella *** **strain W12-1067**

**Name**	Peg Nr.*	**Copies**	**Feature**	**Closest homolog**
ISFw1	1, 2	1	DDE_4 SF	ISRin1, can. *Regiella insecticola*
ISFw2	129		DDE_Tnp_1, IS4,	IS4, *Nitrosomas sp*.
ISFw3	100, 130, 195, 232, 260, 274, 556, 647, 738, 756, 758, 896, 930, 965, 1146, 1212, 1298, 1415	18	Pfam_rve, integrase	IS481, *wHa_02240 Wolbachia endosymbiont* of *Drosophila simulans*
ISFw4	277	1	Tra8, IS30, integrase	*Fphi_0709*
ISFw5	331	1	transposase, partial	*Fphi_0985*
ISFw6	568	1	DDE_4_2	*Aasi_1822, can. Amoebophilus asiaticus* 5o2
ISFw7	822	1	DDE_3, transposase ISFtu1	FTH_0348, *Francisella holarctica* OSU18
ISFw8	895	1	DDE_4_2	*Aasi_1822, can. Amoebophilus asiaticus* 5o2
ISFw9	1118	1	transposase, partial	*Fphi_1490*
ISFw10	1214	1	DDE_Tnp_1, IS4/5	*Fphi_0257*
ISFw11	1295	1	DDE_4_2, partial	OTT_1632, *Orientia tsutsugamushi* str. Ikeda
Tp	701	1	DDE_Tnp_1_2, truncated	*WRi_008070 Wolbachia* sp. wRi
Tp	898	1	HTH_Tnp_IS630,	ISRin2, ORFA, can. *Regiella insecticola*
Tp	1255	1	IS4	*NE061598_00570 F. tularensis* NEO

*see Additional file 2: Table S1.

#### Virulence factors and secretion systems

A number of genes involved in *Francisella* pathogenesis have been identified in various different studies, including *in vivo* negative selection screens of transposon mutant libraries of *Francisella* strains [41-44], reviewed in [45] and [46]. We therefore looked for homologs of these virulence genes within the genome sequence of *Francisella* strain W12-1067. Some of them are shown in Table 4, and others, like LPS, capsule, type IV pili or FPI-associated genes, are described below. Various known virulence factors of *Francisella* exhibit homologs in strain W12-1067. For the most important virulence factors of *F. tularensis*, encoded by the genes of the *Francisella* Pathogenicity Island (FPI), no close homologs could be identified within the genome. However, we identified an FPI-like island at the genome sequence which will be discussed later.

**Table 4:** (Putative) virulence factors of strain W12-1067

**Name**	**Peg Nr.**	**Feature**	**Closest homolog**
MglA	693	Macrophage growth locus protein	FTN_1290 (82%)
CapBCA	74-76	Capsular biosynthesis	Fphi_1486-88 (nd)
FeoB	1092	Ferrous iron transport protein	FTN_0066 (82%)
PilT	1248	Twitching motility protein	FTN_1622 (94%)
DeoB	802	Phosphopentomutase	FN3523_1666 (80%)
BipA	325	GTP-binding protein	Fphi_0048 (91%)
SurA	543	Peptidyl-prolyl cis-trans isomerase	FTN_0559 (69%)
MviN	843	Flippase	FTN_0276 (61%)
HlyB	1149	Toxin secretion ABC transporter	FTN_1693 (74%)
Phospholipase	743	Lecithinase/hemolysin	FTN_0436 (75%)
Lysophospholipase	167	Lysophospholipase	Fphi_1625 (67%)
HlyC/CorC	182	CBS domain, putative hemolysin	FTN_1006 (80%)
ClpB	1114	ClpB chaperone domain	FTN_1743 (89%)
Chitinase 1 (372 aa)	490	GH18_chitinase-like superfamily	Fphi_0209 (83%)
Chitinase 2 (590 aa)	816	Chitinase_glyco-hydro_19 domain, PP location, CBM	Fphi_0512 (69%)
Chitinase 3 (437 aa)	818	Chitinase_glyco-hydro_19 domain, SP	Fphi_0512 (70%)
Chitinase 4 (979 aa)	1009	GH18_chitinase-like superfamily, SP, two internal repeats	FTW_0142 (55%)
Chitinase 5 (731 aa)	1044	GH18_chitinase-like superfamily, SP, EC location, 2 × CBMn	FN3523_1814 (49%)
Chitinase 6 (843 aa)	1477	GH18_chitinase-like superfamily, SP, EC location, 2 × CBM	Fphi_0208 (55%)
Hypothetical protein	523	Ankyrin repeat, Ank_2 domain	---
Hypothetical protein	567	Ankyrin repeat, Ank_2 domain	---
Hypothetical protein	1109	Ankyrin repeat, Ank_4 domain	---
Hypothetical protein	173	TPR domain	FTW_0991 (63%)
Hypothetical protein	1024	TPR domain	Fphi_0624 (69%)
Hypothetical protein	1030	TPR domain, TPR_16	FTM_1557 (72%)

SP, signal peptide; EC, extracellular; PP, periplasmic; CBM, carbohydrate binding motif; TPR, tetratricopeptide repeat.

Interestingly, the genome of isolate W12-1067 exhibit six different chitinases of different sizes and putative location (cytoplasmic, periplasmic or extracellular) (Table 4), which was in good agreement with the phenotypically identified chitinase activity within the supernatant of *Francisella* W12-1067 cells (see Additional file 1: Figure S1C). Four chitinases (peg_818, 1009, 1044 and 1477) exhibit a signal peptide and therefore could be secreted by the general secretion system (Sec). Two of the chitinases (peg_0816 and peg_0818) are separated by a gene encoding a putative DNA-invertase (peg_0817). We could not identify a homolog of this protein in the available genome sequences of *Francisella* strains. We were unable to determine whether the invertase is involved in DNA inversion in *Francisella* and whether this may influence the expression of the nearby chitinase genes. Chitinase peg_0816 is 76% identical to chitinase peg_0818. Both chitinases exhibit a homolog in *F. philomiragia* 25017 (Fphi_0512) and 25015, but not in the other *Francisella* strains, whereas homologs of the chitinases 1, 4 and 5 could be found in the genomes of *Ft. tularensis*, *F. holarctica* and *F. philomiragia*.

Chitinases (FTN_0627 and FTN_1744) have been detected as essential virulence factors of *Ft. novicida* and for biofilm formation [42,44,47]. In *L. pneumophila* a chitinase was also found to be involved in the infectivity of *Legionella* for mice [48]. It would be interesting to further analyze the role of the different chitinases in strain *Francisella* W12-1067.

Furthermore, we identified three hypothetical proteins (peg_523, 567 and 1109) containing ankyrin-repeat domains. These proteins did not exhibit significant homology to any known protein (Table 4). For *L. pneumophila* it was shown that ankyrin-repeat-containing proteins are involved in the pathogen–host interplay during intracellular replication [49,50].

Furthermore, we looked for genes encoding putative antibiotic resistance proteins. We identified ten putative multidrug resistance proteins (peg_126, 152, 681, 682, 683, 764, 1134, 1430, 1444 and 1445). In addition, we identified a Chloramphenicol acetyltransferase (peg_183), a Chloramphenicol phosphotransferase (peg_1413) and a Streptomycin-6 kinase (peg_1416) without a homolog in any of the available *Francisella* genomes. We performed growth inhibition experiments with the isolate W12-1067 and found that levels of resistance to erythromycin, chloramphenicol and streptomycin were comparable to those of *F. philomiragia* (data not shown). We also identified a putative Acriflavin resistance protein (peg_810) exhibiting 85% amino acid identity to the AcrB protein (Fphi_1007) of *F. philomiragia* 25017.

Since we identified putative signal peptides at the N-terminus of some of the chitinases and chitinase activity within the supernatant, we searched the genome sequence for genes of the general Sec system. The identified proteins are given in the Additional file 4: Table S2. We identified all proteins necessary for a putative functional Sec system plus two signal sequence recognition proteins (SRP) and three different signal peptidases. Therefore, strain W12-1067 seems to encode a functional Sec system for the transport of proteins across the inner membrane. We could not detect genes encoding homologs of a type-II secretion system (T2SS), but proteins (HlyB, HlyD, TolC2) of a putative T1SS (Table S2). We were also able to identify a putative functional Tol-Pal system generally involved in vitamin B12 or colicin translocation, but also in capsule synthesis and outer membrane vesicle formation [51,52]. The presence of a putative T6-like SS will be discussed in the next section.

#### A new putative homolog of the Francisella pathogenicity island (FPI) and regulatory proteins

T6SS are widely distributed amongst diverse Gram-negative species. It is a complex molecular machine which injects effector proteins to target cells or bacteria [53]. T6SS are involved in virulence and in eukaryotic cell targeting. They are also reported to have antibacterial activity [54,55]. Most systems are able to function “anti-eukaryotically” or “anti-bacterially”, and one system has been reported to be able to do both [53,56,57]. It was also proposed that T6SS may target and defend predatory eukaryotes in the environment [58]. For an overview see [53].

**Figure 5:**
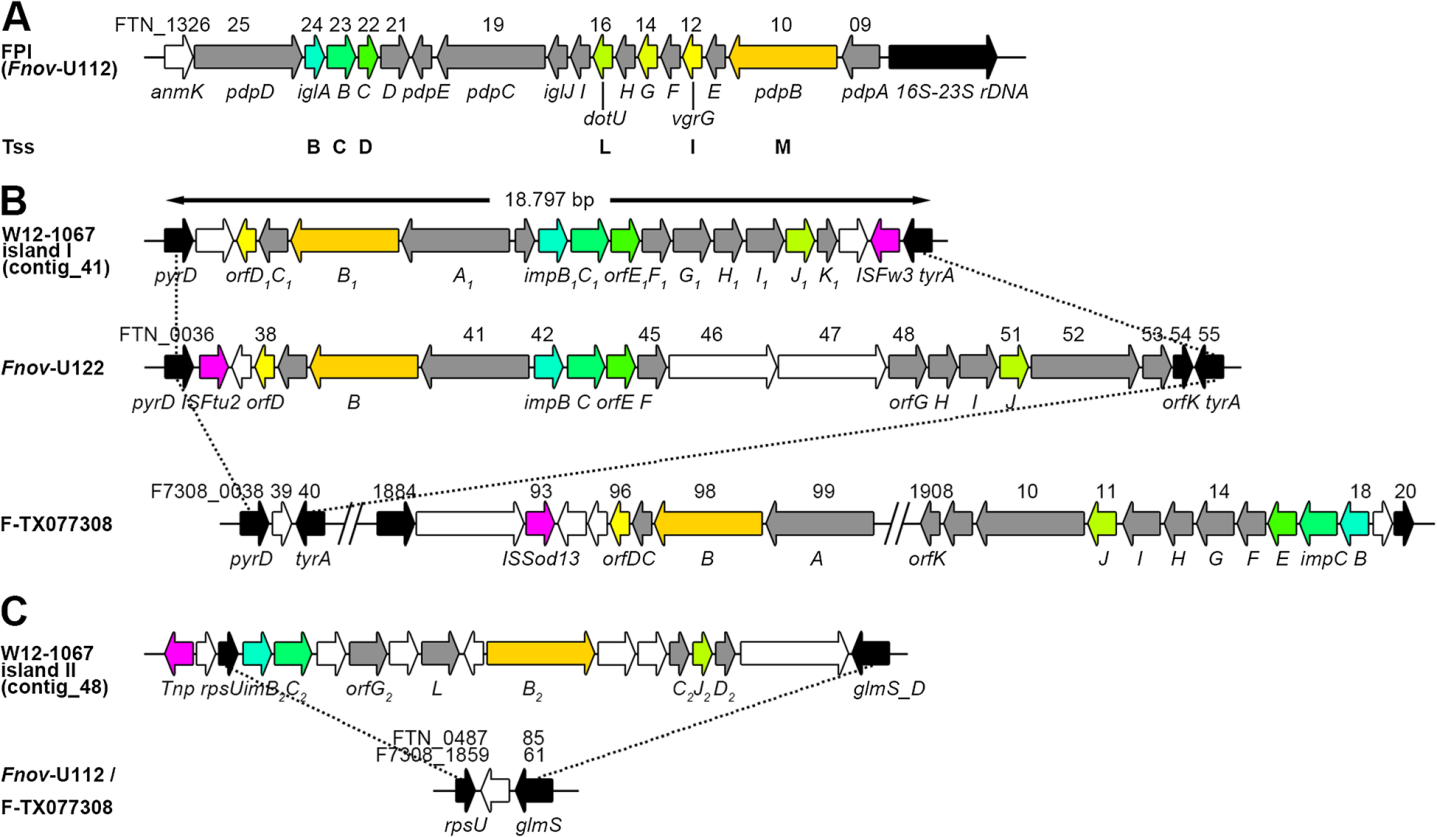
**Genetic organization of T6 secretion systems in *Francisella*.** Organization of the FPI island of *Ft. novicida* U112 **(A)** and of genomic islands I **(B)** and II **(C)** encoding putative T6-like secretion systems of *Francisella* isolate W12-1067. Genomic island I is integrated between genes *pyrD* and t*yrA* in W12-1067 and *Ft. novicida* U112, whereas it is integrated between F7308_1884 und F7308_1920 in *Francisella* strain TX077308 (F-TX077308). Genomic island II is integrated between *rpsU* and *glmS* in W12-1067 and is not present in *Ft. novicida* U112 and *Francisella* strain TX077308. Genes (ORFs) are indicated by arrows. Gene names are given below the genes, and the protein-encoding gene (peg) numbers are given in Table 5. Genes encoding homologous proteins are boxed in the same color, with the exception of pink (mobile elements) and black (conserved core genome genes).

*Francisella* strains exhibit an FPI encoding a functional T6SS, needed for preventing phagolysosomal fusion and escape into the cytosol, which is therefore essential for intracellular replication and pathogenicity of *Francisella*[59-63]. The island is ~33 kb in length, encodes for 15–19 open reading frames (ORFs) and is present in *Ft. tularensis* strains, *Ft. novicida* and *F. philomiragia* (Figure 5A). In strains of *F. tularensis* this island is duplicated [36,64-66]. However, it is still unclear yet whether both copies are needed for full virulence of these strains. Genes of the locus were named *igl* (intracellular growth locus, [66]) and *pdp* (pathogenicity determinant proteins, [65]). The complete locus was identified in 2004, and it seems to be acquired via horizontal gene transfer because of a lower G + C content compared with the core genome [60,64,65].

We were not able to identify a close homolog of the FPI within the genome of strain W12-1067. However, we identified two FPI-like clusters which seem to encode a further putative T6SS (Figure 5). Clusters I (Figure 5B) and II (Figure 5C) each code for 16 ORFs. Homologs of *iglABC*, *dotU* and *pdbB* are present, and the whole island I is similar to an FPI-like element (FTN_0038-0054) recently identified within the genome of *Ft. novicida*, but not yet discussed further [60,64]. Within the genome sequence, island I is also localized between the *pyrD* and *tyrA* genes and island II between *rpsU* and *glmS* (Figure 5B and C). All genes, features of the ORFs and closest homologs of both systems identified within the genome of W12-1067 are given in Table 5. In a recently published model of an FPI-encoded T6SS-like apparatus IglAB (TssBC), IglC (TssD), DotU (TssL), PdpB (TssM) and VgrG (TssI) were identified as core proteins of the T6SS [67]. We identified similar proteins within the identified loci of strain W12-1067. In strain F-TX077308, the FPI-like gene cluster is localized at a different site within its genome sequence (Figure 5B). Altogether these findings led us to hypothesize that these loci may encode a further T6-like SS in *Francisella* strain W12-1067. As mentioned above, infection assays and EM analysis indicateed that strain W12-1067 is able to replicate intracellularly (Figures 3 and 4), which also suggest that there should be a functional T6SS in this strain. However, since the EM analysis did not yet clearly demonstrate whether the strain is released into the cytosol, further experimental data are needed to verify this hypothesis.

**Table 5:** **Genes of the putative T6SSs I and II of *Francisella *** **isolate W12-1067**

**Name (T6SS I)**	**Peg Nr. (aa)**	**kDa**	**Feature**	**Closest homolog (% aa identity)**
OrfD1	950 (92)	9.9	DUF2345, VgrG-like, Rhs family	FTN_0038 (57%)
OrfC1	951 (138)	15.7	SP, lipid attachment site (IglE-like)	FTN_0039 (66%)
OrfB1	952 (1097)	127.1	SP, TM domain (PdpB-like/TssM)	FTN_0040 (58%)
OrfA1	953 (735)	84.6	Cl*	FTN_0041 (63%)
Orf48	954 (48)	5.8	SP	---
ImpB1	955 (179)	20.8	homologous to IglA/TssB	FTN_0042 (96%)
ImpC1	956 (508)	57.7	homologous to IglB/TssC	FTN_0043 (91%)
OrfE1	957 (214)	23.1	helical bundle domain (putative TssD)	FTN_0044 (77%)
OrfF1	958 (356)	41.3	Cl	FTN_0045 (51%)
OrfG1	059 (406)	47.3	Cl, T6SS-associated like protein	FTN_0048 (72%)
OrfH1	960 (274)	31.9	C-terminal TM domain	FTN_0049 (63%)
OrfI1	961 (501)	58.6	Cl	FTN_0050 (72%)
OrfJ1	962 (203)	23.8	DUF2077 (putative DotU/TssL)	FTN_0051 (63%)
OrfK1	963 (126)	13.1	DUF4280	FTN_0054 (82%
Orf374	964 (374)	43.3	Cl	Hyp. protein (33%) Flavobacterium
IglD3	1538 (398)	46.1	DUF876 (TssK)	IglD (49%) F-TX077308
**Name (T6SS II)**	**Peg Nr.**	**kDa**	**Feature**	**Closest homolog (% aa identity)**
ImpB2	1376 (177)	19.9	Cl, homologous to IglA/TssB, DUF770	FTN_0042 (42%)
ImpC2	1375 (493)	55.5	Cl, homologous to IglB/TssC, COG3517	FTN_0043 (47%)
Orf204	1374 (204)	22.4	Cl,	---
OrfG2	1373 (405)	47.1	Cl, IglD-like	F7308_1914 (28%)
Orf368	1372 (368)	43.4	Cl	---
OrfL	1371 (629)	74.6	Hypothetical protein	F7308_1916 (33%)
Orf96	1370 (96)	11.1	2 TM domains	Hyp. protein (49%) *Prevotella histicola*
OrfB2	1369 (928) [1020]	106.3	Cytoplasmic membrane, SP	FTN_0040 (20%)
Orf457	1368 (457)	53.4	Hypothetical protein	---
Orf254	1367 (254)	30.3	Cl	---
OrfC2	1366 (146)	17	SP	FTN_0039 (24%)
OrfJ2	1365 (195)	23.1	DUF2077 (putative DotU/TssL)	DotU, (29%) *Desulfonatronospira*
OrfD2	1364 (111)	12.4	Cl (DUF2345, vgrG)	---
ORF705	1363 (705)	77.7	Cl	---

*determined by using “psortb” (http://www.psort.org/psortb); Cl, cytoplasmic localization; TM, transmembrane domain; SP, signal peptide; (aa), number of amino acids.

Various regulatory proteins are known for *Francisella*, and the regulators MglAB, SspA, PmrA, FevR, MigR and Hfq were identified to be involved in the regulation of genes of the FPI [59,61,68-76]. In *F. tularensis* the genes of the FPI are upregulated during intracellular growth within macrophages [61,68,77-80]. In the genome of strain W12-1067 we identified only homologs of MglAB and Hfq, but also two further response regulator proteins (OmpR1 and R2) as well as two sigma factors (Sig-70 and Sig-32) and homologs of IscR, ArsR, Crp and Fur (see Additional file 5: Table S3). In addition, proteins involved in the stringent response could be identified (SspB, SpoT and RelA).

#### Surface structures: The wbt locus, capsule and type IV pili

(i) **LPS.** The lipid A core portion of the LPS anchors the lipopolysaccharide structure to the outer membrane, whereas the O-polysaccharide chain is the predominant epitope recognized by the immune system and specifies antigenicity. *Ft. tularensis* subspecies-specific antisera have been generated and applied to antigen detection in *F. tularensis*[81]. Furthermore, LPS is used as an antigen in seroprevalence studies and for diagnostic of human tularemia [6,82,83]. However, the LPS produced by *F. tularensis* is less endotoxic compared to other Gram-negative bacteria, such as *E. coli*, a phenotype also known for *L. pneumophila* LPS [84-86]. Genes probably involved in the biosynthesis of the O-polysaccharide chain are given in supplementary Table 4 (see Additional file 6: Table S4). Obviously, there is one cluster of genes (peg_0636-0646) for which homologs were found in *Ft. novicida* and *F. philomiragia*, whereas for genes peg_0609-0615 and peg_0628-0631 the homologs were only found in *F. philomiragia* or *Ft. novicida* U112, respectively. In addition, there is another cluster of genes (peg_0616-0627) which seems to have no homologs in *F. tularensis* or *F. philomiragia*, but homologs were identified in different bacterial species, as in *Vibrio*, *Pseudomonas*, *Sulfurovum* or *Acinetobacter* (see Additional file 6: Table S4). The LPS structure of strain W12-1067 has not yet been analyzed further.

(ii) **Capsule.** Electron microscopy of strain W12-1067 grown on agar plates or in medium revealed the absence of a capsule (Figure 4A and data not shown). However, we identified three ORFs encoding homologs of *capBCA* genes (Table 4). The *capBCA* locus of *Francisella* is similar to determinants encoding the poly-gamma-glutamic capsule in *Bacillus anthracis*[87]. These genes have been shown to be essential for the virulence of *F. tularensis* in a murine model of tularemia [44,87]. Further experiments will be needed to analyze the role and structure of the capsule of *Francisella* strain W12-1067 and to identify conditions necessary for the putative induction of capsule gene expression.

(iii) **Type IV pili.** Electron microscopy of strain W12-1067 did not show any pili on the surface of the bacteria grown in medium at 37°C (Figure 4A) or on agar plates (data not shown). However, we identified homologs of the type IV pilus (Tfp) encoding loci of *F. philomiragia* ATCC 25017 in the genome sequence of W12-1067 (see Additional file 7: Table S5) [88]. We could not identify a PilA homolog, a homolog of the second PilW protein Fphi_0522 and of the two additional PilA/PilE pilus assembly proteins (Fphi_0424/0449). Tfp systems are known to be involved in bacterial pathogenesis, bacterial adhesion and twitching motility [89]. In *L. pneumophila* Tfp pili are required for twitching motility, natural competence, biofilm formation and are involved in the attachment to host cells [90-93]. Tfp have been observed on the surface of *Ft. novicida* and *Ft. holarctica*[94,95], and Tfp are involved in the pathogenicity of *Francisella*[96-98].

#### Toxin–antitoxin systems

We identified three different type II toxin–antitoxin systems (peg_0599-0600, peg_0704-0705 and peg1296-1297). In type II systems, the antitoxin (small unstable protein) sequesters the toxin (stable protein) through protein complex formation (reviewed in [99]). Peg_0599 encodes a protein (84 amino acids, putative toxin), exhibiting a Pfam_Plasmid_Txe domain and 67% amino acid identity to the YoeB toxin of *Pleurocapsa sp*. PCC7319. peg_0600 encodes the respective putative antitoxin (83 amino acids), exhibiting a Pfam_PhdYeFM (type II toxin–antitoxin) and shows 60% amino acid identity to the prevent-host-death protein of *Methylocystis rosea*. The second system is composed of protein Peg_0704 (96 amino acids), exhibiting a HTH-XRE motif and a HigA-antidote (VapI) domain and shows 65% amino acid identity to protein LLO_065 of *Legionella longbeachae* NSW150. The respective putative toxin (HigB, 97 amino acids) is encoded by peg_0705, exhibiting a Pfam_plasmid killer domain, and shows 62% amino acid identity to the plasmid maintenance system killer protein of *Deferribacter desulfurricans* SSM1. Both described systems seemed to have no homolog in the sequences of *Francisella* available so far and are localized on contig_34 of the draft genome of strain W12-1067.

The third system is localized on contig_46. peg_1296 encodes for a protein (85 amino acids, putative antitoxin), exhibiting a Pfam_PhdYeFM domain (type II toxin–antitoxin system), and shows 66% amino acid identity to the plasmid-encoded (pF243) protein F243_0001 of *F. philomiragia* ATCC 25017 and 65% identity to the Phd protein (pFNL10_p3) of *Ft. novicida*. The respective putative toxin (84 amino acids) is encoded by peg_1297, exhibits a Pfam_Plasmid_Txe (YoeB) domain and shows 79% amino acid identity to F243_0002 of *F. philomiragia*. Plasmid pF243 is 5,072 bp long and was predicted to encode six putative ORFs [100]. ORFs F243_0001 and F243_0002 are organized in an operon that is similar to the *phd*-*doc* post-segregation killing system operon of pFNL10 [100,101]. This post-segregation killing mechanism relies on the difference in stability of the antitoxin and toxin. In the daughter cells the plasmid-free bacteria will be killed by the activity of the toxin [102,103]. Chromosomally encoded toxin–antitoxin systems may stabilize chromosomal regions during evolution and seem to be involved in host regulatory networks or fitness advantages [102]. Less is known so far about toxin–antitoxin systems in *Francisella*, but they have been used to construct plasmids which are stable without a selective marker gene [101,104].

## Conclusions

The isolation of strain W12-1067 in Germany indicates for the first time the presence of a close homolog in Europe of the new species *F. guangzhouensis* recently identified in China. In addition, to our knowledge this is the first report of a *Francisella* species other than *F. tularensis* isolated in Germany. Further research is needed to analyze the spectrum of *Francisella* species present in natural habitats in Germany.

The growth optimum of the isolate is approximately 30°C, it is able to grow without additional cysteine within the medium and the strain is halotolerant. The analysis of the genome sequence of the new isolate revealed a lot of known *Francisella* virulence factors, but also the absence of FPI, the major virulence factor of *Francisella* strains. Instead, the isolate seems to exhibit a putative new T6SS, and W12-1067 is able to replicate within eukaryotic host cells. Therefore, the isolate seems to be an interesting species to be analyzed further.

## Competing interests

The authors declare that they have no competing interests.

## Authors’ contributions

KH performed the annotation, the comparative and phylogenetic analysis and drafted most of the manuscript. EB generated the end version of the draft-genome which was submitted to NCBI/GenBank. KR achieved the phenotypic analysis, growth experiments and infection assays. TS performed the MAUVE alignments and generated figures. CL and JF were involved in the isolation and preliminary typing of strain W12-1067. GH performed the EM analysis of strain W12-1067. RG participated in writing the manuscript. All authors read and approved the final manuscript.

## Supplementary Material

Additional file 1: Figure S1 Phenotypic analysis of *Francisella* isolate W12-1067, *Ft. holarctica* strain LVS (Ft. LVS), *Ft. novicida*, *F. philomiragia* and *L. pneumophila* Paris (Lpp). Growth on BCYE agar plates with (+) and without (-) additional cysteine (Cys) (A). The results are representative of three independent experiments. Growth in medium T in the presence of different amounts of NaCl (B). Results are mean standard deviations of three independent experiments of duplicate samples. Chitin degradation by the supernatant of different strains grown in medium T and then incubated on agarose plates containing 0.1% deacetylated glycol chitin (C). Halos around the inoculation site revealed the presence of degrading activity after 2 days of incubation at 37°C. The results represented are representative of three independent experiments. Click here for file

Additional file 2: Table S1 Annotated genes (peg), rRNAs and tRNAs. Click here for file

Additional file 3: Figure S2 MAUVE alignment of strain W12-1067 and *Ft. novicida* U112. The colored boxes represent homologous segments free of genomic rearrangements. Homologous regions are connected by lines between genomes. Non-boxed regions lack homology between genomes. White areas indicate that the sequences are specific to a genome. (The synteny between both genomes was not estimated, since the genome of W12-1067 is a draft genome). Click here for file

Additional file 4: Table S2 Genes of the Sec, type I and Tol secretion systems. Click here for file

Additional file 5: Table S3 Regulatory proteins. Click here for file

Additional file 6: Table S4 The *wbt* gene cluster of strain *Francisella* W12-1067. Click here for file

Additional file 7: Table S5 Type IV pili encoding genes. Click here for file
