# Supplementary figures and images for: Genome sequence and phenotypic analysis of a first German Francisella sp. isolate (W12-1067) not belonging to the species Francisella tularensis
Source: BMC Microbiol. 2014 Jun 25;14:169. doi: 10.1186/1471-2180-14-169 (PMC4230796; doi:10.1186/1471-2180-14-169)

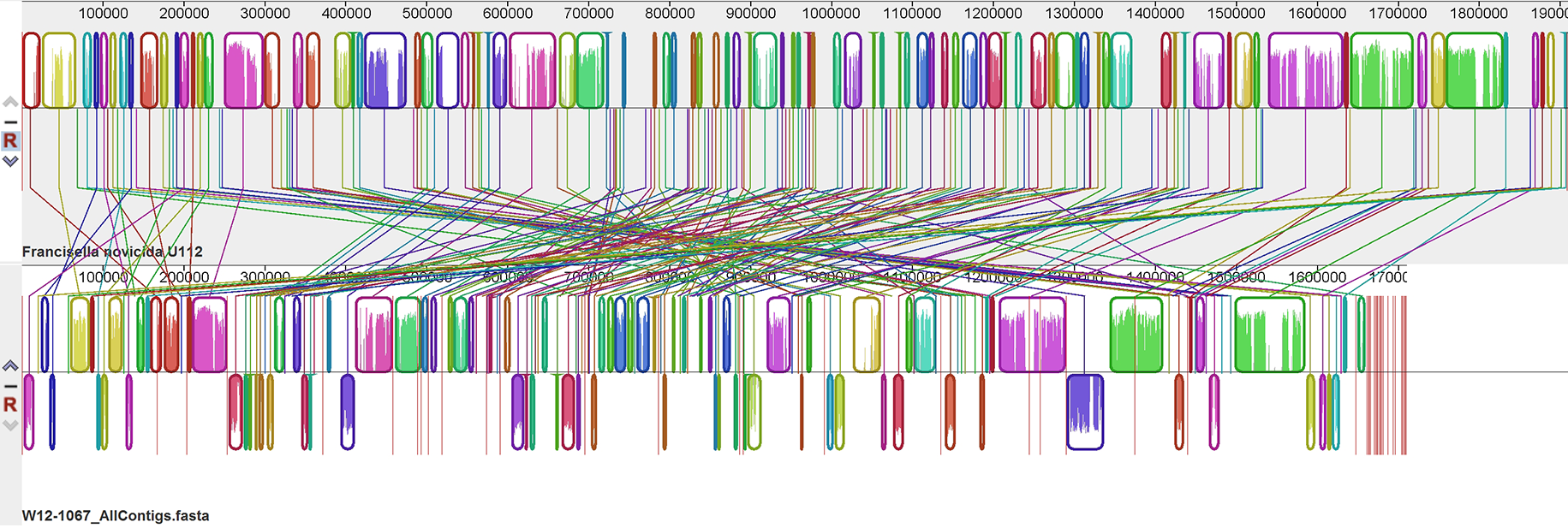

Supplement: Additional file 3: Figure S2 — MAUVE alignment of strain W12-1067 and Ft. novicida U112. The colored boxes represent homologous segments free of genomic rearrangements. Homologous regions are connected by lines between genomes. Non-boxed regions lack homology between genomes. White areas indicate that the sequences are specific to a genome. (The synteny between both genomes was not estimated, since the genome of W12-1067 is a draft genome). [file 1471-2180-14-169-S3.tiff]
