# Supplementary material for: Genome sequence and phenotypic analysis of a first German Francisella sp. isolate (W12-1067) not belonging to the species Francisella tularensis
Source: BMC Microbiol. 2014 Jun 25;14:169. doi: 10.1186/1471-2180-14-169 (PMC4230796; doi:10.1186/1471-2180-14-169)
Supplement: Additional file 4: Table S2 — Genes of the Sec, type I and Tol secretion systems. [file 1471-2180-14-169-S4.docx]

**Table S2** Genes of the general secretion (Sec) system, type I secretion and Tol system proteins

| **Name** | **Peg Nr.** | **Feature** | **Closest homolog**  **(% aa identity)** |
| --- | --- | --- | --- |
| SecA | 394 | secretion system protein A, ATPase | FTN_0672 (89%) |
| SecB1 | 922 | secretion system protein B, chaperone | TX_0111 (81%) |
| SecB2 | 1278 | secretion system protein B, chaperone | TX_0729 (82%) |
| YajC | 467 |  | TX_1600 (81%) |
| SecD | 466 | secretion system protein D, IM component | FTN_1095 (82%) |
| SecF | 465 | secretion system protein F, IM compnent | FTN_1094 (83%) |
| SecE | 787 | secretion system protein E, IM component | 00M_0027 (72%)# |
| SecG | 995 | secretion system protein G, IM component | Fphi_0978 (82%) |
| SecY | 857 | secretion system protein Y, IM component | Fphi_0657 (93%) |
| SRP | 796 | signal recognition protein, FtsY domain | Fphi_1022 (79%) |
| SRP | 323 | SRP domain, PRK10867, SPB domain | Fphi_1770 (89%) |
| LepB | 1330 | signal peptidase I | FN3523_1531 (79%) |
| SppA | 977 | signal peptide peptidase | FTN_0118 (72%) |
| LspA | 737 | lipoprotein signal peptidase | FTN_0440 (80%) |
| TolC1 | 247 | outer membrane efflux protein domain | Fphi_1676 (77%) |
| TolC2 | 1141 | type_I_secretion_TolC | FTN_1703 (79%) |
| HlyB | 1149 | Toxin secretion ABC transporter | FTN_1693 (74%) |
| HlyD | 1150 | HlyD_3 domain, SP, membrane fusion protein | FTN_1692 (80%) |
| TolQ | 1396 | inner membrane protein, Tol-Pal system | OOM_0335 (88%) |
| TolR | 1397 | inner membrane protein, Tol-Pal system | Fphi_470 (83%) |
| TolA | 1398 | inner membrane protein, Tol-Pal system | F7308_0381 (54%) |
| TolB | 1399 | periplasmic/peptidoglycan protein, Tol-Pal | FTN_0355 (74%) |
| Pal |  | outer membrane lipoprotien, Tol-Pal system | FTT_0841 (54%) |

# *F. noatunensis* Toba04; IM, inner membrane; SP, signal peptide; SRP, signal recognition protein; aa, amino acids
