# Supplementary material for: Genome sequence and phenotypic analysis of a first German Francisella sp. isolate (W12-1067) not belonging to the species Francisella tularensis
Source: BMC Microbiol. 2014 Jun 25;14:169. doi: 10.1186/1471-2180-14-169 (PMC4230796; doi:10.1186/1471-2180-14-169)
Supplement: Additional file 5: Table S3 — Regulatory proteins. [file 1471-2180-14-169-S5.docx]

**Table S3 Regulatory proteins**

| **Name** | **Peg Nr.** | **Feature** | **Closest homolog**  **(% aa identity)** |
| --- | --- | --- | --- |
| RpoD (Sig-70) | 180 | RNA polymerase sigma factor | F.gua (97%) |
| RpoH (Sig-32) | 463 | RNA polymerase sigma factor | F.gua (91%) |
| MarR1 | 261 | DNA binding protein | Fn_0936 (74%) |
| MarR2 | 1412 | DNA binding protein | Hyp. protein, (95%)  *Holospora undulata* |
| IscR | 253 | Rrf2_domain | Fn_0936 (73%) |
| OmpR1 | 1329 | response regulator protein | Fn_1532 (91%) |
| OmpR2 | 1342 | response regulator protein | Fphi_1222 (85%) |
| BaeS (PmrB) | 1341 | response sensor protein | Fphi_1221 (96%) |
| ArsR | 1455 | transcriptional regulator | Fphi_1293 (76%) |
| Crp/Fnr | 150 | transcriptional regulator, oxidative_stress | --- |
| Fur | 1168 | ferric uptake regulation protein | FTCG_1680 (88%) |
| Hyp. protein | 607 | HTH_XRE | Hyp. protein (59%)  *Holospora undulata* |
| LysR family protein | 589 | transcriptional regulator | FTN_0392 (63%) |
| Hfq | 449 | RNA binding protein | Fphi_1538 (93%) |
| MglA (Sspa) | 693 | Macrophage growth locus protein | FTN_1290 (82%) |
| MglB | 694 | SspB super family, | Fphi_1396 (78%) |
| SpoT | 77 | GDP diphosphokinase, (p)ppGpp | FTN_1198 (81%) |
| RelA | 1272 | GDP pyrophosphokinase | FTN_1518 (81%) |
|  |  |  |  |

Hyp., hypothetical; aa, amino acids
