# Supplementary material for: Genome sequence and phenotypic analysis of a first German Francisella sp. isolate (W12-1067) not belonging to the species Francisella tularensis
Source: BMC Microbiol. 2014 Jun 25;14:169. doi: 10.1186/1471-2180-14-169 (PMC4230796; doi:10.1186/1471-2180-14-169)
Supplement: Additional file 7: Table S5 — Type IV pili encoding genes. [file 1471-2180-14-169-S7.docx]

**Table S5** Type IV pili encoding genes

| **Name** | **Peg Nr.** | **Feature** | ***Fphi* locus tag*** |
| --- | --- | --- | --- |
| PilD | 187 | T4 prepilin leader peptidase | Fphi_1587 |
| Pil | 219 | T4 pilus assembly protein | Fphi_0010 |
| PilN | 220 | T4 pili associated protein | Fphi_0009 |
| PilO | 221 | Type IV pili glycosylation | Fphi_0008 |
| PilP | 222 | T4 pili lipoprotein | Fphi_0007 |
| PilQ | 223 | T4 pili secretion component | Fphi_0006 |
| PilW | 420 | T4 pilus assembly protein | Fphi_1689 |
| PilB | 481 | T4 pili ATPase | Fphi_0117 |
| PilC | 482 | T4 pili inner membrane protein | Fphi_0118 |
| PilA1/E1 | 578 | T4 pili fiber bilding block protein | Fphi_0422 |
| PilA2/E2 | 579 | T4 pilus assembly protein | Fphi_0423 |
| PilE | 1095 | T4 pilus assembly protein | Fphi_0763 |
| PilT | 1248 | Twiching motility protein | Fphi_0996 |
| PilW | 1283 | T4 pilus biogenesis/stability | Fphi_1136 |
| PilZ | 584 | T4 pili | Fphi_0438 |
| FimT | 1519 | T4 pilus assembly protein | Fphi_0157 |
| PilA | --- | T4 pilin protein | Fphi_1748 |
| Hypothetical protein | 749 | T4 pili glycosylation protein | Fphi_0407 |

*Siddaramappa et al. 2012
